# Wrist‐worn actigraphy in agitated late‐stage dementia patients: A feasibility study on digital inclusion

**DOI:** 10.1002/alz.13772

**Published:** 2024-03-18

**Authors:** Ta‐Wei Guu, Anna‐Katharine Brem, Christopher P. Albertyn, Pooja Kandangwa, Dag Aarsland, Dominic ffytche

**Affiliations:** ^1^ Department of Old Age Psychiatry Institute of Psychiatry Psychology and Neuroscience, King's College London London UK; ^2^ Division of Psychiatry Departments of Internal Medicine China Medical University Beigang Hospital Yunlin Taiwan; ^3^ Sleep Medicine Center and Mind‐Body Interface Laboratory (MBI‐Lab) China Medical University Hospital Taichung Taiwan; ^4^ University Hospital of Old Age Psychiatry, University of Bern Bern Switzerland; ^5^ Centre for Age‐Related Medicine Stavanger University Hospital Stavanger Norway; ^6^ National Institute for Health Research (NIHR) Maudsley Biomedical Research Centre (BRC) at South London and Maudsley NHS Foundation Trust London UK

**Keywords:** Actigraphy, BPSD, compliance, dementia, feasibility

## Abstract

**BACKGROUND:**

Wrist‐worn actigraphy can be an objective tool to assess sleep and other behavioral and psychological symptoms in dementia (BPSD). We investigated the feasibility of using wearable actigraphy in agitated late‐stage dementia patients.

**METHODS:**

Agitated, late‐stage Alzheimer's dementia care home residents in Greater London area (*n* = 29; 14 females, mean age ± SD: 80.8 ± 8.2; 93.1% White) were recruited to wear an actigraphy watch for 4 weeks. Wearing time was extracted to evaluate compliance, and factors influencing compliance were explored.

**RESULTS:**

A high watch‐acceptance (96.6%) and compliance rate (88.0%) was noted. Non‐compliance was not associated with age or BPSD symptomatology. However, participants with “better” cognitive function (*R* = 0.42, *p* = 0.022) and during nightshift (F_1.240, 33.475 _= 8.075, *p* = 0.005) were less compliant. Female participants were also marginally less compliant (F_1, 26 _= 3.790, *p* = 0.062).

**DISCUSSIONS:**

Wrist‐worn actigraphy appears acceptable and feasible in late‐stage agitated dementia patients. Accommodating the needs of both the patients and their carers may further improve compliance.

## 1 BACKGROUND

Measuring behavioral rhythmicity and specific patterns of activities using wearable devices such as an actigraphy watch (wrist‐worn actigraphy) is receiving more attention in ageing and dementia research because these devices are capable of objectively and longitudinally capturing behavioral changes,^1^, ^2^, ^3^ Certain circadian rhythmicity changes are potential markers of ageing or progression of dementia,^1^, ^4^ and linked to the development of behavioral and psychological symptoms of dementia (BPSD).^5^ Specific BPSD, such as agitation and aggression (A/A), are associated with the severity of dementia,^6^ heavily burdensome to caregivers and society, and detrimental to the patients themselves, while difficult to treat.^7^, ^8^

Although using actigraphy as an objective tool to assess behavioral patterns in healthy older adults^1^ and early stages of dementia without agitation appears feasible and acceptable in some studies,^9^, ^10^ its application in late‐stage agitated dementia patients is still largely unknown. A recent scoping review provided an informative roadmap, where most of the nine studies included were proof of concept in design, aiming to characterize and/or monitor agitated behaviors in dementia patients.^11^ Only two small studies (*n* = 1 and *n* = 5, respectively)^12^, ^13^ recruited patients with well‐defined significant agitation at baseline, and no study reported acceptance and compliance rates in detail, leaving the feasibility and acceptability of wearable devices over an extended period questionable. A recent systematic review found poor acceptance of assistive technology including wearable devices in older people living in long‐term care facilities,^14^ and that acceptance and compliance could worsen with the severity of cognitive impairment. Another review suggested that a personalized strategy is needed for both the patients and their caregivers, because with worsening cognitive function, increasing support to wear the watch from caregivers may also be needed.^15^ We therefore investigated acceptance (whether the participant agrees implicitly or explicitly to wear the watch) and compliance (time spent wearing the watch) in late‐stage dementia patients with significant agitation over 4 weeks, and explored from both patients and caregivers the potential reasons and solutions for non‐acceptance and non‐compliance.

## 2 METHODS

### 2.1 Participants and study procedures

We tested the use of wrist‐worn actigraphy within the Sativex for the treatment of the Agitation & Aggression in Alzheimer's Dementia (STAND) trial. The full protocol of the STAND trial has been published earlier.^16^ Briefly, the STAND trial recruited care home residents aged between 55 and 95 years old with a probable Alzheimer's Disease diagnosis^17^ and clinically significant A/A symptoms, as defined by the Cohen‐Mansfield Agitation Inventory (CMAI) ≥ 45^18^ and/or Neuropsychiatric Inventory Nursing Home Version (NPI‐NH) agitation sub‐score ≥ 4.^19^ There was no restriction on mobility status; however, care home residents with severe, unstable or poorly controlled medical illness as deemed by the study doctor or the principal investigators, were excluded.

The first participant was recruited on the October 19 2021, and the recruitment continued until July 2022 through the National Institute for Health Research (NIHR) Maudsley Biomedical Research Centre Care Home Research Network (CHRN), and from caregivers or family members responding to trial adverts. After receiving written and witnessed informed consent from either the participant (if deemed to have mental capacity), a personal legal representative, or a professional legal representative. The research team arranged a screening visit to record the demographic and clinical characteristics and to confirm eligibility. The participant was randomized to either the Sativex or placebo arm after passing the screening, and watch‐wearing compliance was examined for 4 consecutive weeks. The team remained blinded throughout the study, and all participants were asked to wear the actigraphy watch on their non‐dominant wrist. Participants and care home staff were informed about the purpose of the watch and instructed to avoid removing or interacting with it. Each participant was visited three times during the trial period (baseline, midpoint, and 4‐weeks trial end) to mount, change, and retrieve the watch, and to obtain trial‐related information.

**RESEARCH IN CONTEXT**

1. **Systematic review**: We searched PubMed for studies applied wearable device to monitor behavioral symptoms in late‐stage dementia patients and found most of the previous studies were in proof‐of‐concept stage, with very small sample sizes and/or short device‐wearing duration. Many studies didn't recruit patients with pre‐defined behavioral symptoms and didn't report detailed compliance data.
2. **Interpretation**: Our study, recruiting the most late‐stage dementia patients with significant pre‐defined agitation at baseline, found that wrist‐worn actigraphy could be a feasible behavior‐monitoring tool for an extended period of time. Unlike in early‐stage dementia patients, we found late‐stage dementia patients’ compliance appears to be better along with the worsening of cognitive function. Clinical setting and sex may also contribute to compliance, but not age or behavioral symptomatology.
3. **Future directions**: The findings would encourage the application of wrist‐worn actigraphy in late‐stage agitated dementia patients of larger scales, to explore the interaction between their behavioral symptoms, circadian rhythm, sleep, and cognitive decline.

### 2.2 Wrist‐worn actigraphy

The “GeneActiv Original,” is one of the most widely‐used research grade wearable tri‐axial accelerometers for the assessment of physical activity and sedentary behavior.^20^ It is worn with a wrist strap but has a blank face that does not indicate the time. Its technical reliability and capacity to classify activity levels have been validated and described in detail previously.^21^ The sampling frequency of the watch was set at 50 Hz. In addition to movement, a light sensor captures environmental light data that can be used for further analysis (Figure S1).

### 2.3 Questionnaires

In late‐stage dementia where verbal communication is impaired, standard cognitive tests are of limited usefulness. We therefore used three questionnaires that are suitable in severe dementia to assess (1) functional capacity (Functional Assessment Staging Tool, FAST^22^), which has validated correlation to cognitive function in late‐stage dementia^23^; (2) BPSD symptoms and their severity (Neuropsychiatric Inventory‐Nursing Home version, NPI‐NH)^19^; and (3) a specific scale for the severity of agitation/aggression (Cohen‐Mansfield Agitation Inventory, CMAI).^18^

### 2.4 Interviews and feedback

At each visit, participants and their caregivers were briefly interviewed to collect qualitative feedback on their feelings and attitude toward the watch‐wearing. As most of the participants were expected to have severe dementia and may not have been able to respond verbally, their non‐verbal responses such as hand gestures, facial expressions and other body languages were also noted.

At baseline, the initial response to wearing the actigraphy watch was recorded. Previous watch‐wearing habit was checked with both the participant and caregiver. At the midpoint and the trial end (fourth week), participants were interviewed again for their feelings and experiences wearing the watch. Their primary caregivers were interviewed separately for their opinions on assisting participants to wear the watch and for any observable behavior indicating non‐acceptance or non‐compliance (including watch damage or removal). If any significant non‐acceptance or non‐compliance was observed, the caregiver was additionally asked for the context, potential reasons, and suggestions for improvement.

### 2.5 Data analysis

The R markdown package developed by Activinsight^21^ was used to generate a daily personal activity report to visually inspect specific shift periods of non‐wear (i.e., whether the non‐wear fell within day shift 06:01‐14:00, evening shift 14:01‐22:00, or night shift 22:01‐06:00). The non‐wear duration was calculated using a well‐validated, open‐sourced R package GGIR (version 2.8‐2)^24^, ^25^ and compared with the visually inspected results.

In the study, acceptance rate was defined as the percentage of participants who explicitly or implicitly (e.g., did not try to remove) accepted the watch. The compliance rate of each participant was calculated as the percentage of total wear time (trial duration—nonwear time/ trial duration × 100). Correlations between demographic and clinical parameters at baseline (including age, FAST score, NPI and CMAI total scores and sub‐scores; FAST score 6a to 6e and 7a to 7f were converted to 6.0 to 6.8 and 7.0 to 8.0 for ordinal regression) and watch wear compliance were calculated with Spearman's correlation coefficients. Because this is an exploratory study interested in the overall pattern of findings rather than their statistical significance, we did not correct for multiple comparisons when correlating clinical and demographic measures with watch measures. Separate mixed model repeated measure analyses of variance (ANOVAs) were used to investigate the effect of shift (morning, evening, and night shift) and sex (male and female) on non‐compliance across the 4 weeks of the study. Greenhouse‐Geisser correction was used for violations of sphericity. Basic R packages and the SPSS statistical software version 26 (IBM SPSS) were used. *P* < 0.05 was used as the threshold for reporting.

## 3 RESULTS

### 3.1 Quantitative acceptability and compliance

Table 1 shows the demographic and clinical characteristics of the study group. All participants were categorized as moderately severe or severe dementia, and the majority were more severely agitated than the inclusion threshold.

**TABLE 1:** Demographics, clinical characteristics, and compliance of participants.

Demographics	Statistics
Sex, *n* (%)	
Male	15 (52%)
Female	14 (48%)
Age, mean (range, SD)	80.8 (60–92, 8.2)
Ethnicity, *n* (%)	
White	27 (93.1%)
Black/Asian/other	2 (6.9%)

Baseline clinical characteristics	Statistics
FAST score, *n* (sub scores)	
6 (6a, 6b, 6c, 6d, 6e)	9 (1, 1, 1, 2, 4)
7 (7a, 7b, 7c, 7d, 7e, 7f)	20 (15, 2, 1, 2)
Mean NPI‐NH total scores (range, SD)	42.3 (4–100, 25.7)
Total disruptiveness score	11.1 (0–33, 9.5)
Mean NPI‐NH sub‐scores (range, SD)	
Delusion sub‐score	3.9 (0–12, 0.9)
Hallucination sub‐score	3.1 (0–12, 4.3)
Agitation sub‐score	9.2 (0–12, 3.3)
Depression sub‐score	2.6 (0–12, 4.3)
Anxiety sub‐score	3.0 (0–12, 5.0)
Euphoria sub‐score	1.0 (0–8, 2.1)
Apathy sub‐score	3.6 (0–12, 4.1)
Disinhibition sub‐score	2.8 (0–12, 4.2)
Irritability sub‐score	7.4 (0–12, 4.5)
Aberrant motor activity sub‐score	5.8 (0–12, 5.5)
Appetite sub‐score	0.4 (0–6, 1.4)
Nocturnal symptom sub‐score	5.0 (0–12, 4.5)
Mean CMAI total scores (range, SD)	83.9 (42–167, 29.8)

Compliance	Statistics
Overall mean compliance (range, SD)	88.0% (7%−100%, 24.2%)
Week 1 Mean Compliance (range, SD)	85.8% (9.5%−100%, 23.4%)
Week 2 Mean Compliance (range, SD)	86.0% (0%−100%, 31.3%)
Week 3 Mean Compliance (range, SD)	88.7% (13.1%−100%, 22.4%)
Week 4 Mean Compliance (range, SD)	91.4% (0%−100%, 24.2%)

Abbreviation: CMAI, Cohen‐Mansfield Agitation Inventory; FAST, Functional Assessment Staging Scale; NPI‐NH, Neuropsychiatric Inventory Nursing Home Version; SD, standard deviation.

The overall acceptance for wearing the actigraphy watch was 96.6%, and no watch was lost. Out of the 29 participants in the STAND trial, only 1 male participant refused to wear the watch. Most participants accepted the actigraphy watch without difficulty at the first encounter. Although 22 participants were not able to express their attitude or feelings verbally upon the initial encounter, they would nod their head, smile, or give a thumb up to the researcher. This acceptance was stable throughout the trial.

The first four participants (No. 01, 03, 04, and 05) from three different care homes joined the trial before the temporary closure of many care homes in the greater London area associated with the omicron variant coronavirus disease 2019 (COVID‐19) pandemic. As shown in Table 2, watch damage and reduced acceptance and compliance were noticed in this phase, and especially during the first week of the watch‐wearing period. We refer to this reduced period of acceptance as the “first week phenomenon.” To explore this phenomenon, we examined the initial data from these few participants to improve the study conduction, and created an instruction document (Figure S2) to better communicate with the participants and their caregivers.

**TABLE 2:** Participant concerns related to wearing the actigraphy watch at baseline and over the course of the trial.

No.	Sex	FAST	Initial response	Week 2	Week 4
01	F	6e	Accepted wearing the watch. But broke the watch on the fourth day when being assisted for bathing.	Well accepted the device.	Accepted the watch.
03	M	6c	Wore his personal watch on the right (dominant) wrist but accepted to wear the trial watch on the left wrist.	Well accepted the device.	Accepted the watch.
04	M	6b	Refused to wear the watch.	Refused to wear the watch.	Refused to wear the watch.
05	M	7a	Broke the watch on the second day due to discomfort. The care staff put back the broken watch with a bandage and tape on the fifth day. Participant kept wearing it after this but on the other wrist.	Accepted and was able to wear the new watch.	No issue reported by caregivers. The second watch remained intact.
06	F	6d	Very agitated and confused, needed assistance from caregivers to put on the watch.	Accepted the watch.	Accepted the watch.
09	M	7a	Shouted at the researcher when daytime napping was disrupted, needed assistance from family.	Accepted the watch.	Accepted the watch.
11	F	7a	Questioned the need to wear the watch.	Initially insisted the watch belongs to her.	Accepted the watch.
14	F	6a	Questioned the need to wear the watch.	Still questioned the need.	Felt relieved.
25	F	6d	Felt counterintuitive to wear two watches (trial and personal watch) at the same time, also disliked the appearance of the trial watch.	Felt confused when wearing both the trial and personal watch.	Felt relieved as she wouldn't need to wear two watches anymore.

Abbreviation: FAST, Functional Assessment Staging Scale.

**FIGURE 1:**
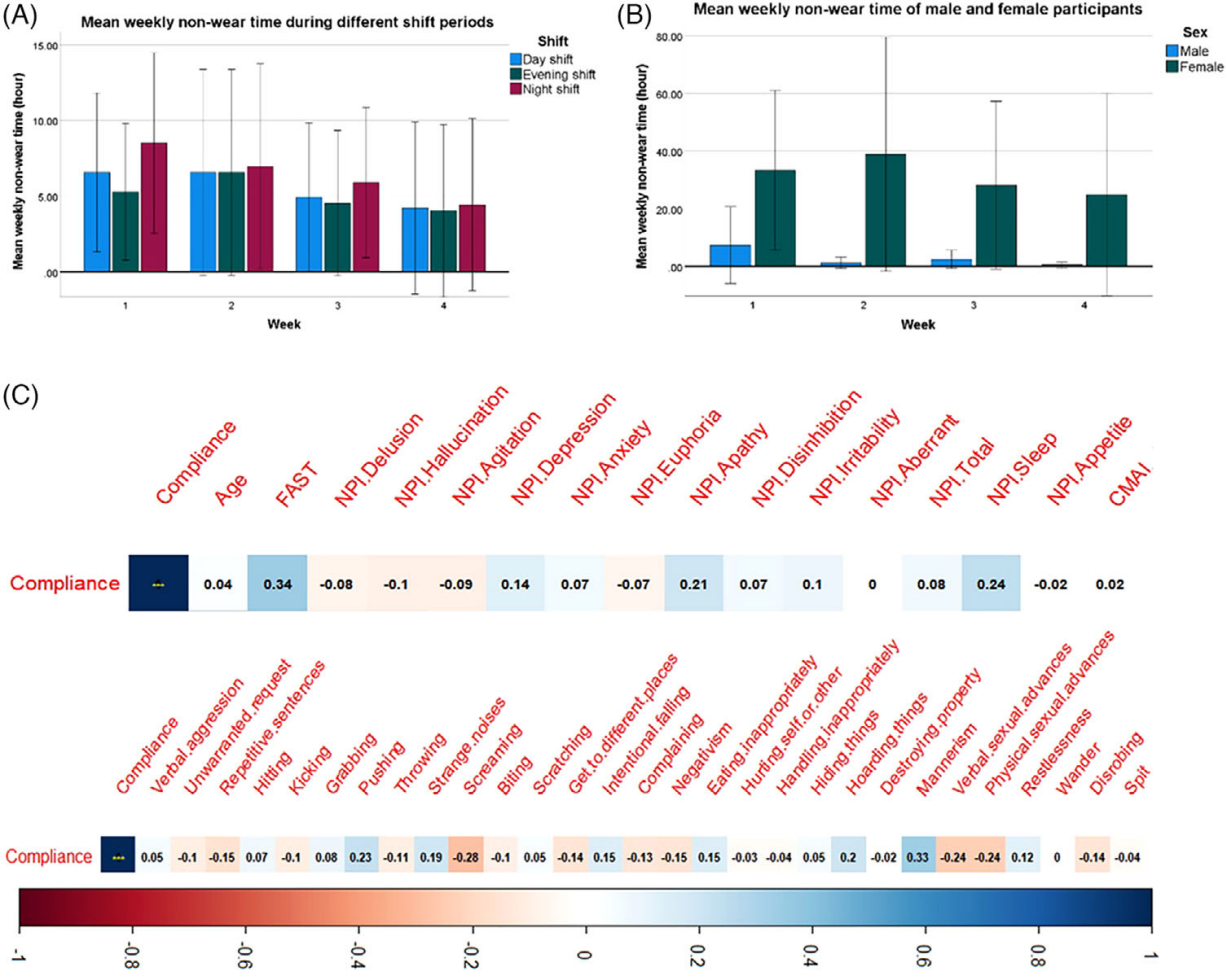
(A) Mean weekly non‐wear time (hours) in different shift periods. (B) Mean weekly non‐wear time (hours) of male and female participants. Error bars indicate the 95% confidence interval. (C) Correlograms showing the Spearman's correlation coefficients (the numbers in the squares) between the compliance and all clinical variables, including the NPI total and sub‐scores (first row) and CMAI sub‐scores (second row); the color scale (from dark blue to dark red) represents the correlation coefficient from 1.0 to −1.0. CMAI, Cohen‐Mansfield Agitation Inventory; FAST, Functional Assessment Staging Tool; NPI, Neuropsychiatry Inventory‐Nursing Home Version.

As shown in Table 1, the average compliance was 85.8% in the first week and 91.4% in the fourth week, although there was no significant effect of week on compliance. Both the research team and caregivers continued to observe participants challenging the need to wear the watch mainly in the first week, but there was no further watch damage occurred after the provision of the instruction document. The non‐compliant (non‐wearing) hours between different shift periods and different sexes are shown in Figure 1A,B, respectively. In the shift‐week model, the shift effect was significant (F_1.240, 33.475_ = 8.075, *p* = 0.005), but the week effect was not (F_1.240, 33.475_ = 1.122, *p* = 0.337). Comparing non‐compliance in different shift periods of the day revealed participants were most likely to be non‐compliant during the night shift (non‐compliance rate 13.6%), followed by day shift (12.0%), and least non‐compliant in evening shift (11.2%). For all weeks combined, non‐compliance in the night shift was significantly higher than non‐compliance in the day shift (*p* = 0.038) or evening shift (*p* = 0.016). There was also a trend level significance of sex difference that female participants were less compliant (F_1, 26 _= 3.790, *p* = 0.062). There was no shift‐week interaction (F_1.511, 40.792 _= 2.039, *p* = 0.153) or sex‐week interaction (F_2.236, 58.126 _= 0.755, *p* = 0.488).

An exploratory correlation analysis between watch‐wearing time and all the demographic and clinical characteristics of the participants shows no specific demographic characteristic, neuropsychiatric symptom, or agitation type (as rated by NPI‐NH or CMAI total or sub‐scores), correlate with compliance even at the lenient level of uncorrected significance used (*P* < 0.05), as in Figure 1C. However, a moderate degree of positive correlation with trend‐level significance was noted between compliance and FAST score (*R* = 0.35, *p* = 0.066), suggesting participants with greater cognitive impairment might have better compliance. This association became more significant if the single participant who refused to wear the watch was included as a totally non‐compliant participant with 0 hour as wear time (*R* = 0.42, *p* = 0.022, see Figure S3).

### 3.2 Qualitative responses from the participants and caregivers

Most participants accepted the actigraphy watch and were able to wear it throughout the study. A minority of participants, mostly females, who expressed concerns or complaints at the initial encounter are summarized in Table 2, along with their follow‐up.

The participant who refused to wear the watch (No. 04) could only express “I don't like to wear it.” His caregiver believed this was associated with his personality. Although caregivers were not sure about his pre‐morbid personality, they reported him to be stubborn and not willing to compromise in their facility. The only male participant who broke the watch (No. 05), couldn't explain the reason why he did so. However, his caregiver believes as he no longer regularly wore a watch, it could be awkward and uncomfortable for him to wear one again initially. When he gradually re‐adapted to this habit, he was able to fully adhere to the protocol. This pattern was also observed in three of the female participants who were not able to express their needs verbally (No. 01, 06, and 11). The caregivers reported difficulty assisting and reminding them in the first week but not in the later weeks.

Two other female participants, both with moderately severe dementia as measured by FAST (participant No. 14, FAST score 6a, and participant No. 25, FAST score 6d), expressed their concerns and complaints when first asked to wear the watch. The caregivers reported that she kept taking off the watch before going to bed despite being advised to keep wearing it, as she felt the watch would interfere with her sleep. A similar behavioral pattern was observed in participant No. 25, who felt confusing to wear two watches and that her personal watch would have to move to her right (dominant) hand. She also complained that the actigraphy watch did not look feminine and stated that she usually took off her watch before going to bed. She took off both watches before bed and often did not replace the actigraphy watch despite being reminded. The care home staff suggested a more feminine looking device with a time reading function might be helpful to solve the problem.

Other than these participants, assistance from caregivers to put on the watch was hardly needed, and most caregivers felt there was no difficulty to remind the participant to keep wearing it. Caregivers felt the instruction document a helpful tool to remind themselves, their colleagues and family visitors of the function and purpose of the actigraphy watch and to avoid unnecessary removal especially during the first week when the device is new to both the participants and caregivers. In addition, although the document advised caregivers to remove the watch for a short while if the participants appeared uncomfortable, most caregivers reported they rarely found the participants to be in discomfort or attempted to remove it. As we used the “on button press mode” which allowed the recording to stop and restart by pressing a hidden button on the watch, participants and caregivers could potentially stop the recording by pressing the button by mistake. However, no accidental button‐pressing was noticed when the device recordings were extracted, and the data stored in every watch were analyzable.

## 4 DISCUSSION

In our study, passive‐wearing of an actigraphy watch appeared to be well accepted by late‐stage dementia patients with significant agitation, and most of them were compliant and able to wear it continuously with minimal assistance from the care home staff during the 4‐week study period, without damaging the watch or interrupting the watch recording.

Participants in advance‐stage dementia often lack the mental capacity to make decisions. In our study, participants were all consented by their “personal legal representative” or “professional legal representative”; however, we still asked for the participants’ own opinions and observed their initial responses when providing the actigraphy watch. This additional step was useful to establish a basic relationship and potentially enhanced the overall compliance. Also, an instruction document to remind the participants, communicate the study needs with caregivers was also believed to help improve overall compliance.

In addition to the “first‐week phenomenon,” we also found the participants were most likely to be non‐compliant during night shift than other shifts. This could potentially result from a common scenario in various clinical settings, where reduced attention,^26^ operation speed, and performance are experienced by night‐shift caregivers, resulting in suboptimal patient care or even increased patient safety issues.^27^ However, the great reduction of nocturnal non‐compliance time (from mean 8.5 to 4.4 h weekly) may imply that with an appropriate adaptation period, even late‐stage dementia participants are still able to gradually establish wearing routine and need less assistance from the caregivers to wear the watch at night.

We did not find significant correlation in our exploratory analysis between compliance and specific neuropsychiatric symptom or agitation measures. The lack of correlation between overall compliance and neuropsychiatric symptomatology is encouraging for future study and practice, and may be explained by the fact that watch‐wearing per se is both a common experience and a neutral stimulus that would not provoke specific emotional nor behavioral reaction; therefore, most participants would be able to keep wearing it, regardless of their symptom severity or type.

On the other hand, even with help from care home staff, female participants appeared to be slightly less compliant than male participants, as reflected in both quantitative and qualitative information. Care home staff found it difficult to encourage specific female participants to keep wearing the actigraphy watch, and attributed this difficulty to the masculine appearance of the watch and a lack of time‐reading function. This implies that a watch is such a familiar item that even in late‐stage dementia the lack of a watch face is recognized as an anomaly and contributes to reduced compliance. Unlike other device factors such as their perceived usefulness and user friendliness (low technical demand) identified in previous studies,^14^, ^15^ we could not find previous studies discussing how the appearance of a watch or wearable device more generally may impact its acceptance or compliance, especially in the context of sex difference. An actigraphy watch with a gender‐neutral appearance and/or a variety of straps allowing participants to choose their preferred option, could possibly enhance participants’ motivation to wear it and, therefore, should be considered by device manufacturers as an option to include in future dementia studies. On the other hand, the time‐reading function is intentionally discarded by many research‐grade actigraphy watches to minimize the potential impact of time checking to the activity measure and to prolong use by reducing battery demand. However, as found in ours and in previous studies,^10^ participants and caregivers suggested it would increase people's willingness to keep wearing the actigraphy device if it also functions as a real watch.

**FIGURE 2:**
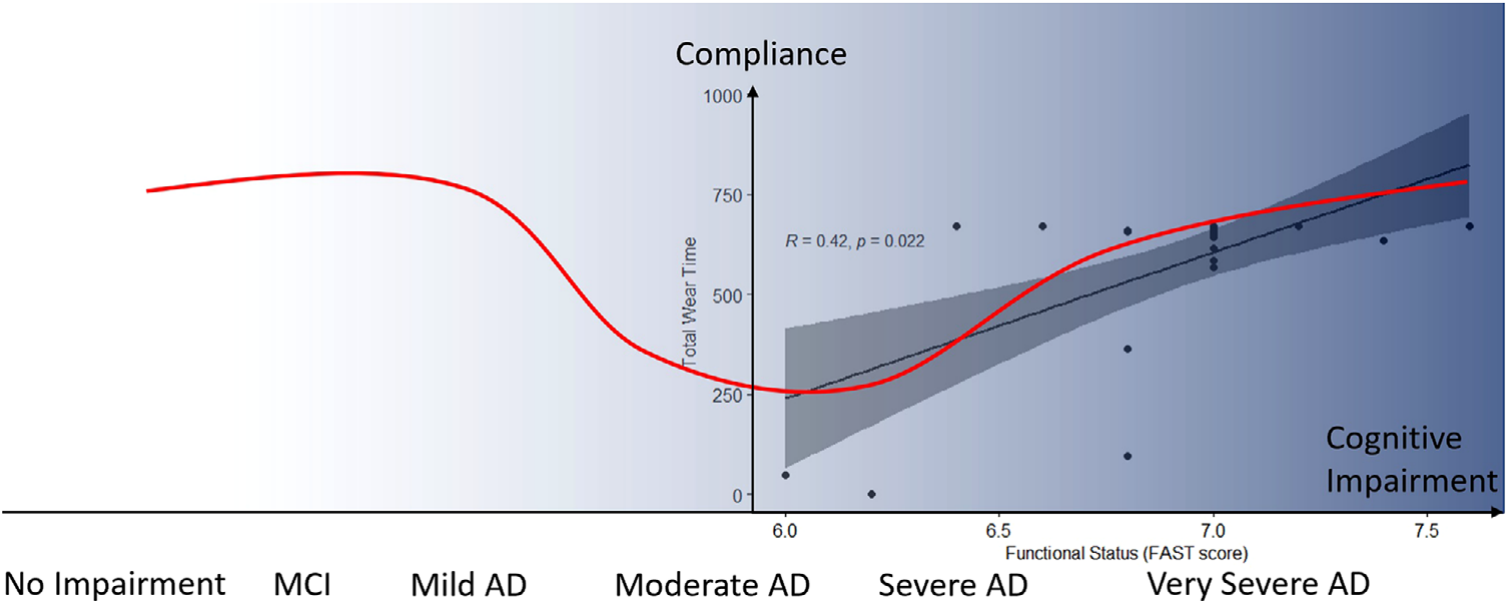
The red line illustrates the theoretical relationship between cognitive function and device‐wearing compliance based on previous studies in elderly participants with better cognitive function and data reported here in late‐stage dementia (the data points and regression line) Passive device‐wearing compliance in elderly participants may follow a “U curve” that participants with both normal and severely impaired cognitive function have the best compliance. The background shading illustrates the progression stage of cognitive decline (darker shading = more advanced dementia).

Cognitive decline has been found to influence acceptance and compliance in previous studies of elderly participants. Study participants with cognitive function in the normal to mild dementia range have overall good compliance.^10^, ^28^ However, studies including participants with mild to moderate dementia found compliance decreased with participants’ cognitive ability,^29^, ^30^ which is contrary to our finding that participants with the worst cognitive function as indicated by FAST score had better compliance. The marginal sex difference found in our study may partly explain the contradictory results as the two female participants with the worst compliance had better functionality/cognition (FAST score 6a and 6d, respectively). However, another interpretation would be that for an actigraphy watch requiring only passive wearing, compliance may follow a U‐shaped curve with respect to cognitive function, as shown in Figure 2. Normal cognitive elderly participants adhere well to the study protocol; compliance declines as participants are unable to follow the protocol or are resistive to change due to their cognitive capacity; finally, participants categorized as severe dementia, as in our study, are much less curious and sensitive to new stimuli such as a watch or watch‐like object and, therefore, comply with wearing the device.

Compared with studies using wrist‐worn actigraphy to monitor agitation in dementia identified in the recent scoping review,^11^ our study recruited the most late‐stage dementia participants with significant pre‐defined agitation at baseline, and has an extended wear time period. Since measuring BPSD in this frail population is very challenging,^8^ the feasibility of using actigraphy established in our study paves the way for future studies to gain a deeper understanding of BPSD and to provide precise measurement‐based treatments.

The study also has limitations. First, our participants only needed to wear the device passively. As poor cognitive function may impair acceptance and compliance in studies with more complex protocols,^29^, ^30^ our results may not be generalizable to studies involving greater device‐related cognitive demands (e.g., remembering to press buttons or record data). Also, this study was done within the STAND trial, which tested the feasibility of using Sativex to reduce agitation in late‐stage dementia so that watch acceptance and compliance might be contaminated by the effect of the investigational product. However, judging from the good initial reaction observed and positive feedback from caregivers, it is likely the wearable device would be similarly feasible in a study without the investigational medicinal product.

In summary, we found wearing a wrist‐worn actigraphy watch is acceptable for late‐stage dementia patients living with significant agitation in care homes, and demonstrates the feasibility of using such devices for behavioral monitoring in this population. Caregivers felt more comfortable assisting and encouraging participants to keep wearing the device when clear and clinically relevant instructions were easily accessible. The type of symptoms or severity of BPSD did not significantly influence compliance in our study, but participants with “better” cognitive ability (in the context of severe dementia) and during night shift appeared less compliant. The marginal sex difference in compliance could reflect the usability and appearance of the device, and these factors should be considered in future studies and watch manufacturer designs.

## CONFLICT OF INTEREST STATEMENT

The authors declare no conflicts of interest. Author disclosures are available in the Supporting information.

## Supporting information

Supporting Information

Supporting Information
