# Supplementary material for: Wrist‐worn actigraphy in agitated late‐stage dementia patients: A feasibility study on digital inclusion
Source: Alzheimers Dement. 2024 Mar 18;20(5):3211–8. doi: 10.1002/alz.13772 (PMC11095432; doi:10.1002/alz.13772)

**Supplementary Figure 1.**

Appearance of the GeneActiv Original actigraphy watch when being worn. A light sensing window is on one side of the front surface (indicated by the yellow arrow), and a hidden button was next it. The watch has no time-reading function.

**
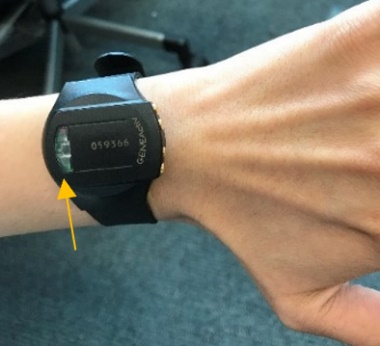
**

**Supplementary Figure 2.**

The instruction document used to better communicate the needs of the study and to enhance compliance.


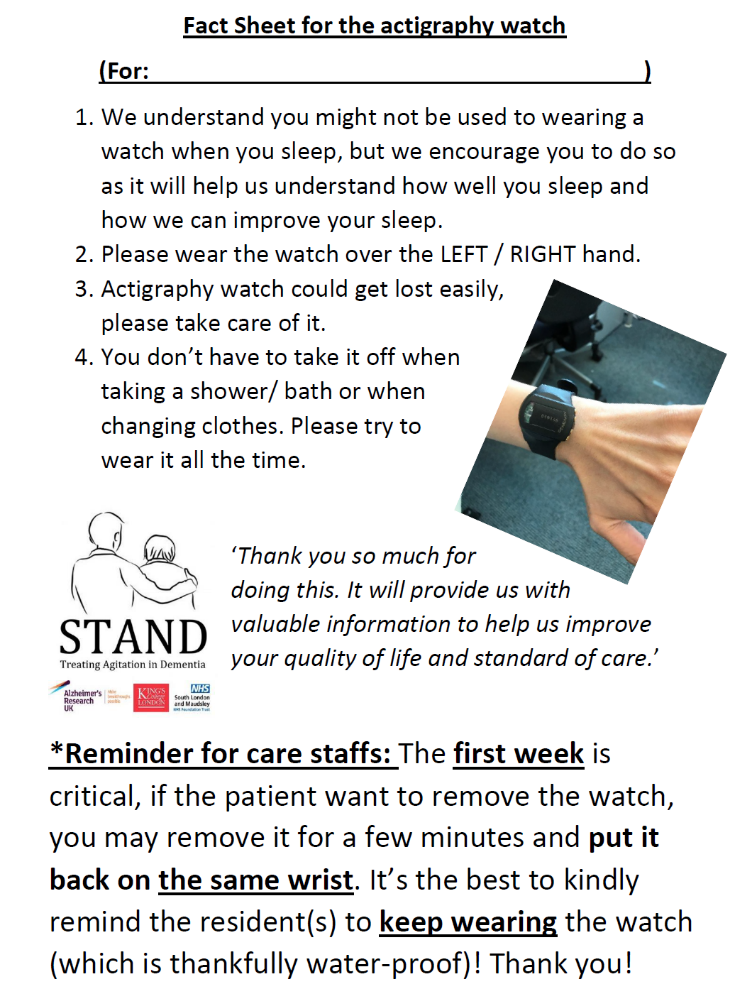


**Supplementary Figure 3.**

Correlation between FAST score and compliance (total watch-wearing time in the trial period) excluding (left) and including (right) the single male participant who refused to wear the watch (indicated by the arrow, the participant’s total wear time was assigned the value 0 minute).


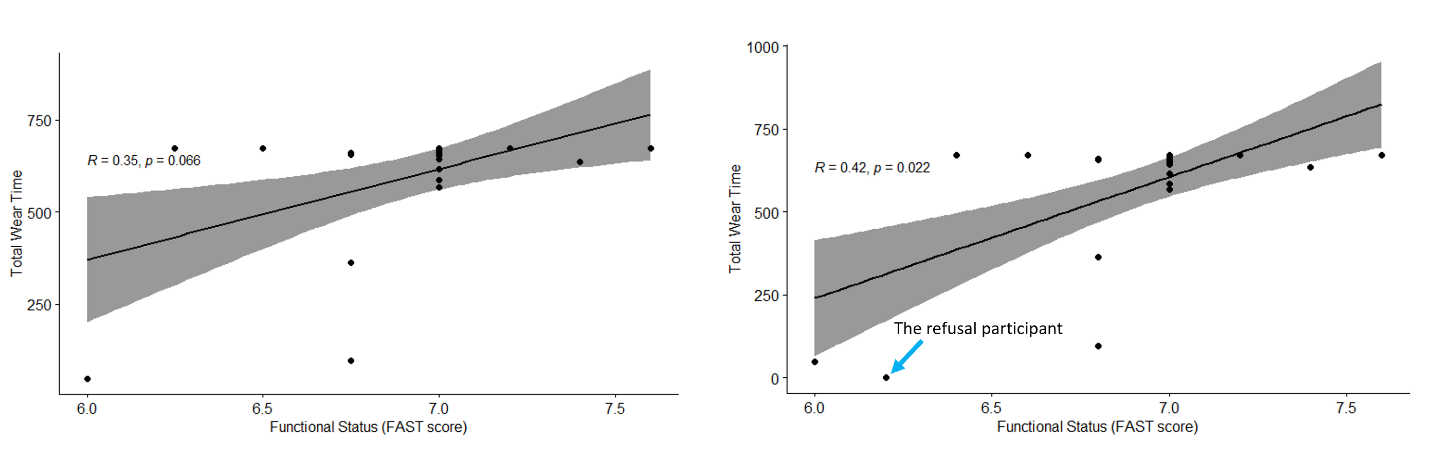

Supplement: Supplementary file 1 — Supporting Information [file ALZ-20-3211-s001.docx]
